# Fur Activates the Expression of *Salmonella enterica* Pathogenicity Island 1 by Directly Interacting with the *hilD* Operator *In Vivo* and *In Vitro*

**DOI:** 10.1371/journal.pone.0019711

**Published:** 2011-05-06

**Authors:** Laura Teixidó, Begoña Carrasco, Juan C. Alonso, Jordi Barbé, Susana Campoy

**Affiliations:** 1 Departament de Genètica i de Microbiologia, Facultat de Biociències. Universitat Autònoma de Barcelona, Bellaterra, Spain; 2 Area de Microbiología, Facultad de Medicina, Universidad de Oviedo, Oviedo, Spain; 3 Centro Nacional de Biotecnología, Consejo Superior de Investigaciones Científicas, Madrid, Spain; University of Osnabrueck, Germany

## Abstract

Previous studies have established that the expression of *Salmonella enterica* pathogenicity island 1 (SPI1), which is essential for epithelial invasion, is mainly regulated by the HilD protein. The ferric uptake regulator, Fur, in turn modulates the expression of the *S*. *enterica hilD* gene, albeit through an unknown mechanism. Here we report that *S. enterica* Fur, in its metal-bound form, specifically binds to an AT-rich region (BoxA), located upstream of the *hilD* promoter (*P_hilD_*), at position -191 to -163 relative to the *hilD* transcription start site. Furthermore, in a *P_hilD_* variant with mutations in BoxA, P_hilD*_, Fur·Mn^2+^ binding is impaired. *In vivo* experiments using *S. enterica* strains carrying wild-type *P_hilD_* or the mutant variant P_hilD*_ showed that Fur activates *hilD* expression, while *in vitro* experiments revealed that the Fur·Mn^2+^ protein is sufficient to increase *hilD* transcription. Together, these results present the first evidence that Fur·Mn^2+^, by binding to the upstream BoxA sequence, directly stimulates the expression of *hilD* in *S. enterica*.

## Introduction

*Salmonella enterica* is a bacterial pathogen that causes numerous diseases, ranging from gastroenteritis to systemic infections, in several hosts including humans. Moreover, it is one of the most important pathogens associated with food-borne illness worldwide [1], [2]. An early step in the pathogenesis of non-typhoidal *Salmonella* species involves their ability to penetrate the intestinal epithelium. Invasion is mediated by the presence of a type III secretion system (T3SS), which is encoded on the tightly regulated *Salmonella* pathogenicity island 1 (SPI1) [3]–[5]. The main regulator of SPI1, HilA, directly activates the expression of the *invF* and *prgH* operons, which encode the components of the T3SS apparatus [6], [7]. InvF facilitates the expression of several effector genes on SPI1 and elsewhere in the *S. enterica* genome [8]–[10]. In turn, *hilA* expression is under the control of other transcriptional activators, HilD and HilC, likewise encoded within the SPI1, and RtsA, located elsewhere in the chromosome [11]–[13]. These three transcription factors independently activate not only HilA expression but also each others' and their own, thus comprising a complex feed-forward regulatory loop [14]. Moreover, HilC and HilD can directly activate *invF* independently of HilA [11].

T3SS expression is also modulated by several distinct environmental signals [3], [15], ensuring that the system is available by the time the bacterial pathogen reaches the distal small intestine, where invasion of the epithelial monolayer takes place [5]. For example, it is well known that osmolarity and bile salt concentration influence T3SS production by controlling *hilD* expression through the EnvZ/OmpR and BarA/SirA two-component systems, respectively [16]–[18]. In addition, it has been shown that phosphate or Mg^2+^ and Ca^2+^ concentrations, sensed by the PhoR/PhoB and PhoP/PhoQ systems, respectively, are likewise involved in SPI1 regulation [3], [19]. In both cases, the sensor system controls expression of the SPI1 repressor HilE, which is encoded by a gene located outside of SPI1. It has been suggested that the negative regulatory effects of HilE are exerted by its direct interaction with HilD, such that HilD-mediated activation of *hilA* is prevented [20]. Another protein, H-NS, has also been described to control SPI1. This nucleic-acid-associated protein, binds to the promoter region of *hilA*, *rtsA*, *hilD* and *hilC* genes diminishing their expression [21], [22].

Iron concentration is also associated with T3SS expression [23]. Fe^2+^ is essential for bacterial development [24]–[26] and *S. enterica* is confronted with different free-iron concentrations during its infectious process. Normally, free Fe^2+^ is scarce inside the host due its sequestration by several different cellular mechanisms [25], [27]. However, in the lumen of the small intestine, where dietary iron is mainly absorbed [28], there is abundant free Fe^2+^. Accordingly, it has been reported that several of the *Salmonella* genes that are expressed when iron is scarce remain silent as long as the bacterium is confined the intestinal lumen [29]. The Fe^2+^ concentration is thought to act as a signal that allows the pathogen to sense its location inside the host [23].

The Fur (ferric uptake regulator) protein is the main regulator of iron homeostasis in many bacteria. As a major regulator of gene expression, it not only controls genes involved in iron homeostasis but also ultimately coordinates intracellular iron levels with many other cellular processes [30]. In transcriptional and translational gene fusions, Fur was shown to activate SPI1 expression by increasing the amount of HilD [4], [23]. Recent studies described the ability of Fur to modulate *hilA* expression by negatively controlling the levels of the H-NS global regulator [31]. In the presence of Fur·Fe^2+^, *hns* expression is repressed. The resulting decrease in the H-NS concentration reduces the repression that it exerts on the *hilA* promoter, thus allowing a rise in the expression of this gene [31], [32]. Nevertheless, the previously described Fur·Fe^2+^-mediated activation of *hilD* expression remains unknown [23], [31].

In *Escherichia coli,* Fur exhibits Fe^2+^-dependent DNA-binding activity to a specific sequence, namely the Fur box, located in the promoter region of genes directly repressed by Fur [33]. The Fur box is a 19-bp consensus sequence organized either as two inverted repeats separated by 1-bp, or as at least three contiguous hexamers, 5′-NATWAT-3′ (where N is any nucleotide and W is an A or a T), aligned in either a direct or an inverse orientation [33]–[35]. In the Fe^2+^-bound form, *E. coli* Fur represses genes involved in respiration, flagellar chemotaxis, the TCA cycle, glycolysis, methionine biosynthesis, phage DNA packaging, DNA synthesis, purine metabolism, and redox stress resistance [36]–[39]. Moreover, *E. coli* Fur has also an indirect positive effect on some genes by repressing the expression of the *rhyB* [40]. The absence of RhyB for pairing at the ribosomal binding site of mRNAs of genes positively regulated by Fur prevents their degradation by subsequent recruitment of the RNA degradosome [41].

Fur has been characterized in several other bacterial species [42]–[45] and other Fur-regulated pathways not related with sRNA have been described [46]. For instance, in *Neisseria meningitidis*, the Fur·Fe^2+^ complex has been shown to act directly as a transcriptional activator once it binds to the promoter region of several virulence-associated genes [47]. Other Fur activation pathways have been reported in *Pseudomonas aeruginosa, Yersinia pestis, Helicobacter pylori*, and *E. coli* [48]–[51]. In *S. enterica* two sRNAs, RfrA and RfrB, have been identified. Both are homologous to *E. coli* RyhB and participate in the Fur-mediated positive control of genes such as *sodB* [23], [52]. Nevertheless, neither RfrA nor RfrB mediates Fur control of *hilD* expression [23]. Moreover, direct control by Fur of the SPI1 repressor, *hilE*, has been ruled out [23].

To understand the molecular mechanism(s) that modulate *hilD* expression by Fur·Fe^2+^, we analysed whether the Fur protein of *S. enterica* serovar Typhimurium directly controls *hilD* expression. Our results show that Fur protein, in its metal-bound form, binds to an AT-rich operator located upstream of the *hilD* promoter region (*P_hilD_*), and it acts directly as a transcriptional activator of *hilD*. These findings help to elucidate the role of iron in the regulation of SPI1 expression and provide the first evidence of a Fur-mediated direct activation mechanism in *S. enterica*.

## Materials and Methods

### Bacterial strains, plasmids and growth conditions

All bacterial strains and plasmids used in this work are listed in Table 1. Bacterial cultures were grown at 37°C in LB. When necessary, ampicillin (100 µg/ml), chloramphenicol (34 µg/ml), or kanamycin (150 µg/ml) was added to the bacterial culture. When needed 2,2-dipyrydil (DPD) was added to the medium at a concentration of 0.2 mM [31], [53].

**Table 1:** Bacterial strains and plasmids used in this work.

Strain or plasmid	Relevant features	Source
*Salmonella enterica* strains		
SV5015	*S. enterica* serovar Typhimurium SL1344 His^+^	[83]
UA1875	SV5015 but carring the pKOBEGA TS plasmid; Amp^R^	[81]
UA1880	As SV5015 but Δ*fur::cat;* Cm^R^	[81]
UA1891	As SV5015 but Δ*hilD::kan*; Kan^R^	This study
UA1892	As UA1880 but Δ*hilD::kan*; Cm^R^, Kan^R^	This study
* *UA1888	As SV5015 but *kan*::*P_hilD_*; Kan^R^	This study
* *UA1889	As SV5015 but *kan*::P_hilD*_; Kan^R^	This study
* *UA1890	As UA1880 but *kan*::*P_hilD_*; Cm^R^, Kan^R^	This study
*Escherichia coli* strains		
DH5-α	*supE4* Δ*lacU169* (Ø80 *lacZ*ΔM15) *hsdR17 recA1 endA1 gyrA96 thi-*1 *relA1*	Clontech
BL21(DE3)pLysE	F^−^ *ompT hsdS*_B_ (r_B_^−^m_B_^−^) *gal dcm* (DE3) pLysE (Cm^R^)	Stratagene
Plasmids		
pET15b	His_6_ tag expression vector; Amp^R^	Novagen
pGEM®-T	PCR cloning vector; Amp^R^	Promega
pKOBEGA	*bla P*_BAD_ *gam bet exo* pSC101 oriTS	[84]
pKD4	*bla* FRT *Kan* FRT PS1 PS2 oriR6K	[60]
pUA1111	pGEM®-T with a 337-bp spanning P*_hilD_* (−247 to +90)	This study
pUA1112	pGEM®-T with a 337-bp spanning P_hilD*_ (−247 to +90) bearing mutations in BoxA	This study

### *In silico* searches for Fur binding sites

The 337-bp *P_hilD_* spanning −247 to +90 (relative to the transcription start site [12]) was used for *in silico* searches with the Virtual Footprint online framework program [54]. The searches were carried out using the pre-existing *P. aeruginosa* (16-mer) matrix [54].

### Protein purification

*S. enterica fur* was PCR-amplified using suitable oligonucleotides (Table S1), cloned into the pET15b expression vector, and transformed into *E. coli* BL21(DE3) pLysE strain. The Fur protein was purified using the TalonTM Metal Affinity Resin Kit (Clontech), as reported [55], and eluted from the affinity column by thrombin cleavage in buffer A (50 mM Bis-Tris/borate pH 7.5, 5 mM MgCl_2_, 10% glycerol) containing 500 mM NaCl. Fur was then loaded onto a Q-Sepharose equilibrated with buffer A containing 100 mM NaCl and eluted with a 600–1000 mM NaCl gradient. *S. enterica* Fur, which is free of *E. coli* H-NS protein, is expressed as dimers. The activity of purified *S. enterica* Fur was confirmed by electrophoretic mobility shift assays (EMSAs), which tested the ability of the protein to bind the promoter region of a confirmed Fur-regulated gene, *foxA* [56]. Figure S1 shows the Fur binding region in P*_foxA_* and the EMSA results using purified Fur protein.

### Electrophoretic mobility shift assays

Appropriate DNA probes were obtained by PCR using suitable DIG-labeled oligonucleotides (Table S1). Fur EMSAs were done as previously described, with slight modifications [57]. In each case, 100 ng of each DIG-labeled DNA probe (20 nM) was incubated with increasing concentrations of Fur in buffer B (10 mM Bis-Tris/borate pH 7.5, 5% glycerol, 1 mM MgCl_2_, 40 mM KCl, 100 µg BSA/ml, 0.2 mg salmon sperm DNA/ml) with or without 100 µM MnCl_2_. For competitive assays, at least a 200-fold excess of either specific or non-specific non-labeled DNA was added. To assay the binding ability of Fur in its apo form, EDTA chelator was included in the binding mixture at a concentration of 1 mM. The binding mixture was incubated for 10 min at 37°C, after which the samples were separated by 5.5% polyacryamide gel electrophoresis (PAGE) in Bis-Tris buffer [57]. The DIG-labeled DNA-protein complexes were detected by following the manufacturer's (Roche) protocol.

### Footprinting assay

For the footprinting assay, the 375-bp [α-^32^P]-*Nco*I-*Hin*dII *P_hilD_* DNA (10 nM) from pUA1111 was incubated with increasing concentrations of Fur (1.5–100 nM) for 15 min at 37°C in buffer C (50 mM Bis-Tris/borate pH 7.5, 5% glycerol, 10 mM MgCl_2_, 1 mM MnCl_2_). DNase I digestion was carried out by addition of the enzyme in binding buffer containing 5 mM CaCl_2_ followed by incubation for 5 min at 37°C; the reactions were stopped by the addition of 25 mM EDTA. The samples were ethanol precipitated, resuspended in 6 µl of loading buffer, and fractionated on 6% denaturing (d) PAGE [58]. As the molecular weight marker, a G + A sequence reaction [59] was carried out and run in parallel with the corresponding footprinting reactions.

### Construction of *S. enterica* mutant derivatives

*S. enterica* UA1888 and UA1889 strains, containing a Kan^R^ cassette (inserted at position –192) and either wild-type (*P_hilD_*) or the Fur BoxA mutant variant (P_hilD*_), and the *hilD* knock-out mutant (UA1891) were constructed using the one-step PCR-based gene replacement method as described [60] and the appropriate oligonucleotides (Table S1). The PCR products were transformed in UA1875 carrying the pKOBEGA plasmid (Table 1). In the *P_hilD_* or P_hilD*_ mutant derivative, transcription orientation of the *kan* gene was opposite that of the *hilD* gene, thus avoiding promoter interference. Indeed, the expression of neither *hilD* nor the downstream *prgH* genes was affected by the presence of the Kan^R^ cassette in UA1888, and the expression levels were the same as those obtained using the SV5015 wild-type strain. All constructs were transferred as described [61] into the SV5015 wild-type strain or the null *fur* mutant derivative (Δ*fur*, UA1880) by transduction using the P22int7(HT) bacteriophage and the suitable constructed strain as donor. The absence of the prophage in the transductants was determined by streaking them onto green plates, as described previously [62]. The obtained mutants were verified by PCR, using the appropriate primers (Table S1), and by nucleotide sequencing.

### Quantitative RT-PCR assays

Quantitative real time RT-PCR (qRT-PCR) assays of *hilD* or *foxA* expression in different genetic backgrounds were carried out. To maximize the iron effect, bacteria were grown in LB medium, which contains saturated iron concentrations. When needed and to generate an iron-limiting environment DPD was added to the medium. All bacterial strains were overnight cultured and then diluted 1/100 in the appropriate media (with or without DPD) and incubated aerobically at 37°C. Once the bacterial culture reached OD_550_ = 0.8, the cells were harvested and then the RNA was extracted using the Quiagen RNeasy kit following the manufacturer's instructions. The qRT-PCR assays were performed as previously reported [63] using suitable oligonucleotides (Table S1). It should be noted that the *hilD* oligonucleotide pair is located upstream the Kan^R^ cassette insertion site in the *hilD* knock-out mutant (UA1891), thus allowing determination of the mRNA level in this genetic background. The results were normalized with respect to *recA*, a standard control gene not associated with the Fur regulon [64]. A change in the *recA* expression pattern was not observed in any of the genetic backgrounds assayed in this study (see Figure S2, in which 16S RNA is used as the standard). The relative expression level was defined as the ratio between the expression of *hilD* or *foxA* in each mutant derivative and that observed in the UA1888 strain containing a wild-type upstream Fur promoter region (*P_hilD_*).

### *In vitro* transcription assays

The *P_hilD_* upstream region, spanning −247 to +90 (relative to the transcription start site), with either a wild-type BoxA or BoxA* mutant variant was cloned into pGEM®-T, generating plasmids pUA1111 and pUA1112, respectively (Table 1). *In vitro* transcription assays (run-off transcription) were performed using 10 nM *of Hin*dII-cleaved pUA1111 (containing *P_hilD_*) or pUA1112 (P_hilD*_), or with pGEM®-T plasmid DNA (*P_ν_*) as an unrelated Fur control. The pUA1111 vector was also used as a supercoiled DNA RNAP template. All of the DNAs were pre-incubated with increasing concentrations of Fur protein (1.5–200 nM) for 15 min at 37°C in buffer D (50 mM Tris-HCl pH 7.5, 5% glycerol, 5 mM MgCl_2_. 1 mM MnCl_2_, 2 mM spermidine, 10 mM DTT) in a 25-µl reaction. One unit of *E. coli* RNAP Eσ^70^ holoenzyme (USB, Cleveland) and 0.5 mM of each rNTP (with [α-^32^P]-rUTP) were added. The reactions were incubated for 60 min at 37°C and then stopped by the addition of 15 µl of loading buffer followed by heating to 75°C for 10 min. The *in vitro* generated transcripts from the linear templates, which contained the cloned *P_hilD_* or P_hilD*_, the vector promoter (*P_ν_*), and the supercoiled pUA1111 vector, were separated in 6% dPAGE, visualized, and quantified as described [65].

## Results

### Fur·Mn^2+^ binds *P_hilD_* DNA with high affinity

**Figure 1:**
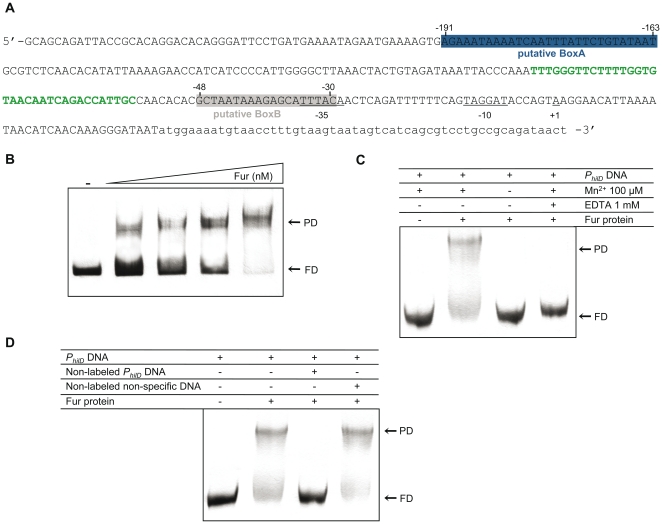
Fur·Mn^2+^ specifically binds *P_hilD_* DNA. **A**. Upstream region (−247 to +90, FrgA) of the *S. enterica* SL1344 *hilD* gene. The putative Fur Boxes A (−191 to −163) and B (−48 to −30), identified through the Virtual Footprint framework program, are boxed in blue and gray, respectively, with the HilD and HilC binding region (−57 to −91 [12]) shown in green. The −35 and −10 promoter (*P_hilD_*) consensus regions and the +1 transcription start site [12] are underlined. The coding region of *hilD* is denoted in lower-case characters. **B**. EMSA of *P_hilD_* DNA (FrgA) (20 nM) in the presence of increasing concentrations (2.5, 12.5, 50, and 187 nM) of Fur protein in buffer B. The mobility of *P_hilD_* DNA in the absence of Fur protein is shown as a control (−). **C**. EMSA of the FrgA probe in the presence of the chelator EDTA or with no Mn^2+^ added to buffer B. The mobility of the *P_hilD_* DNA probe in buffer B containing Mn^2+^ in the absence or presence of 50 nM Fur protein is shown as a negative and positive control, respectively. **D**. EMSA of the FrgA probe in the presence or absence of non-labeled *P_hilD_* or pGEM®-T DNA used as a specific or non-specific competitor, respectively. The specificity of Fur binding was determined usig a 200-fold excess of the corresponding non-labeled DNA. The presence or absence of a component is indicated by + or −, respectively. FD, free *P_hilD_* DNA; PD, the protein-DNA complex.

A search for putative Fur cognate sites in *S. enterica* SL1344 *P_hilD_*, spanning −247 to +90 (relative to the transcription start site [12]), was carried out using the Virtual Footprint online framework [54]. Accordingly, two putative Fur boxes were predicted (Figure 1A): (i) BoxA, at position −191 to −163, corresponding to an AT-rich region (dG + dC content <15%, vs. 50% for the total genome) located ∼ 100-bp upstream of the HilD and HilC binding sites and (ii) BoxB, at position −48 to −30 (dG + dC content ∼31%), overlapping the promoter −35 element and situated ∼ 30-bp downstream from the HilD and HilC binding sites [12] (Figure 1A).

To validate the function of these putative Fur binding sites, the *S. enterica* Fur protein was purified and EMSA studies were performed using the *P_hilD_* region (position −247 to +90) as probe (Figure 1B). All EMSAs were done in buffer containing Mn^2+^ as a common substitute of Fe^2+^ due to its greater stability under aerobic conditions [33], [47]. As shown in Figure 1B and Figure S1, in the presence of Mn^2+^, Fur dimers bound with high specificity and affinity to *P_hilD_*, with an apparent binding constant (K_Dapp_) of about 4.5±1.5 nM, defined as the protein concentration necessary to complex 50% of labeled DNA. In contrast, in the absence of Mn^2+^ or following the addition of EDTA, Fur was unable to form a complex with *P_hilD_* DNA (Figure 1C), suggesting its metal-dependent DNA-binding activity [66]. In addition, the Fur-Mn^2+^ complex displayed a higher DNA affinity than Fur-Mg^2+^ (Figure S1). Together these observations suggest that Fur binds to the *P_hilD_* promoter with the same characteristics as those reported when it acts as a repressor [33].

To test the specificity of the reaction, competition experiments were performed. A large excess (200-fold) of non-specific DNA (pGEM®-T DNA) was unable to compete with *P_hilD_* for Fur·Mn^2+^ binding, whereas an excess of non-labeled *P_hilD_* DNA fully competed for binding with labeled *P_hilD_* DNA (Figure 1D). It is therefore likely that at least one Fur·Mn^2+^ binding site is present in *P_hilD_*, supporting the *in silico* predictions.

### Fur·Mn^2+^ binds to BoxA DNA

**Figure 2:**
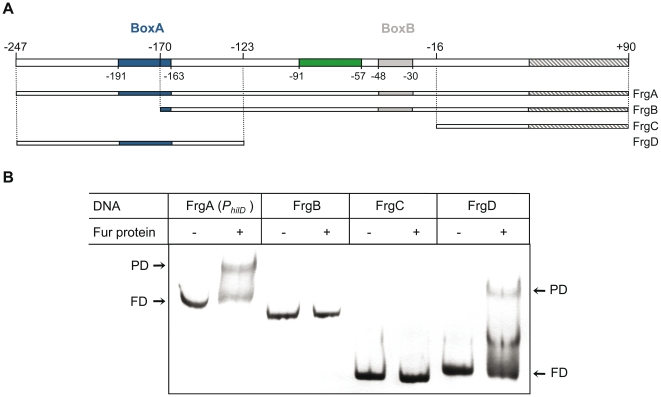
Fur·Mn^2+^ specifically binds BoxA in *P_hilD_*. **A**. Diagram of the *P_hilD_* region. The putative Fur BoxA, located from −191 to −163, and BoxB, from −48 to −30, are framed in blue and gray, respectively. The HilC and HilD binding sites [12], are shown in green. The positions refer to the +1 transcription start site of the *hilD* gene [12]. The *hilD* coding region is striped. **B**. EMSA experiments using FrgA (*P_hilD_*), FrgB, FrgC, and FrgD DNA probes (20 nM) from the *P_hilD_* region in the absence (−) or presence (+) of 50 nM Fur protein.

To elucidate which of the two putative Fur boxes, or even both, binds Fur·Mn^2+^, serial deletions of the *P_hilD_* region were obtained, generating FrgB, FrgC, and FrgD (Figure 2A). As shown in Figure 2B, FrgD (spanning the −247 to −123 interval) but not FrgB (−170 to +90) or FrgC (−16 to +90) bound Fur·Mn^2+^ (Figure 2B). It is therefore likely that: (i) Fur·Mn^2+^ interacts with the region spanning −247 to −123 (containing the putative BoxA, −191 to −163), and (ii) Fur·Mn^2+^ interacts neither with the core *P_hilD_* region nor with the −91 to −57 interval, which includes the HilD and HilC binding sites [12], [21].

### Fur·Mn^2+^ recognizes BoxA DNA and spreads to adjacent regions

**Figure 3:**
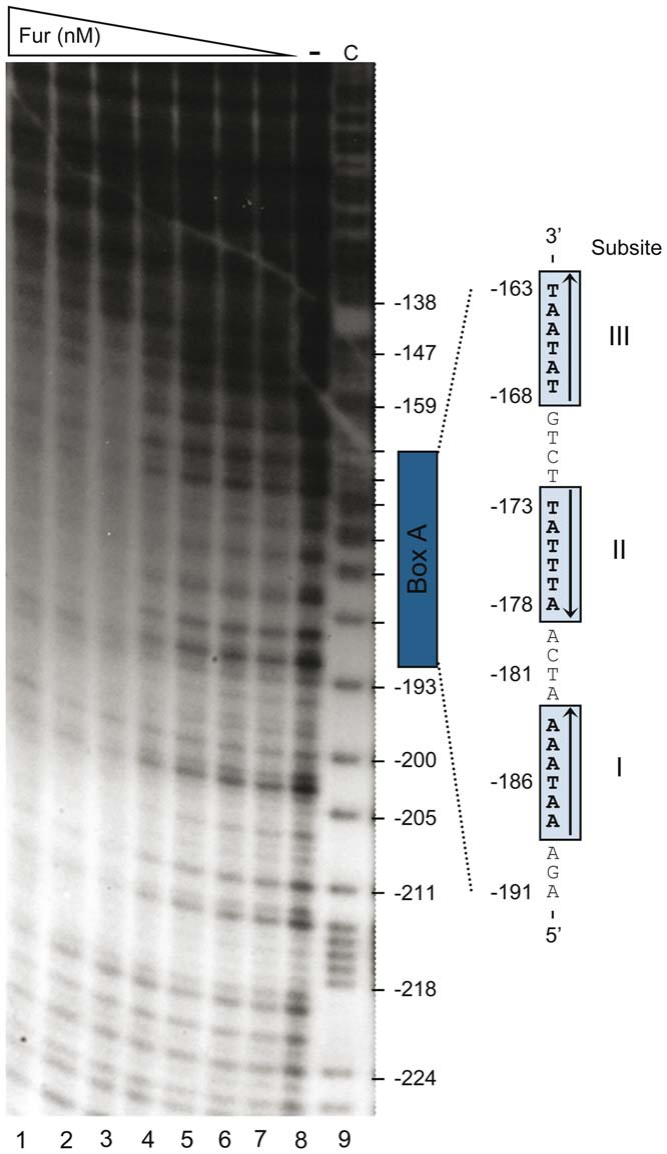
Footprint assay of *P_hilD_* DNA using increasing Fur concentrations. The 375-bp [α-^32^P]-*Nco*I-*Hin*dII *P_hilD_* (top strand) DNA (5 nM) was incubated with increasing Fur concentrations (1.5–100 nM). The positions are related to the transcription start site (+1) [12]. The three BoxA subsites are boxed and enlarged, with arrows denoting their relative orientation. Abbreviations: −, absence of Fur; C, a G + A sequence ladder of the DNA probe was used as molecular size marker.

DNAse I footprinting experiments were carried out to further determine the specific Fur·Mn^2+^ binding site in the *P_hilD_* region. At low Fur·Mn^2+^ concentrations (0.5 Fur dimers/*P_hilD_* DNA), only the −189 to −170 region, including BoxA, was protected from DNase I attack (Figure 3, lane 4). The BoxA site contains three copies of the hexameric 5′-NATWAT-3′ Fur consensus sequence separated from each other by 4-bp. Two hexamers (subsites I and III) are in the direct (→) orientation and one (subsite II) is in the inverse (←) orientation (→4-bp←4-bp→), conforming to a typical Fur box [33]. At limiting Fur·Mn^2+^ concentrations, the protected sequence included subsites I and II spaced by 4-bp, but subsite III was poorly protected (see Figure 3). At sub-saturating and saturating Fur·Mn^2+^ concentrations (2.5∶1 to 10∶1 Fur·Mn^2+^:*P_hilD_* DNA ratios), an extended DNase I -protected interval (from −147 to −219) was observed (Figure 3, lanes 1–3). Sites hypersensitive to DNase I attack were not apparent, suggesting that upon Fur·Mn^2+^ binding no obvious major distortion of the DNA occurred. It is likely that, by cooperative interaction, Fur·Mn^2+^ showed limited spread onto the 5′ (∼ 30-bp) and 3′ (∼ 23-bp) regions of *P_hilD_* DNA (Figure 3). Fur·Mn^2+^, under the concentrations used, halted before reaching the HilD, HilC, RstA binding region (−91 to −57). Similar results were described in *E. coli*, when Fur·Mn^2+^ acts as a transcriptional repressor [33], [67], [68].

**Figure 4:**
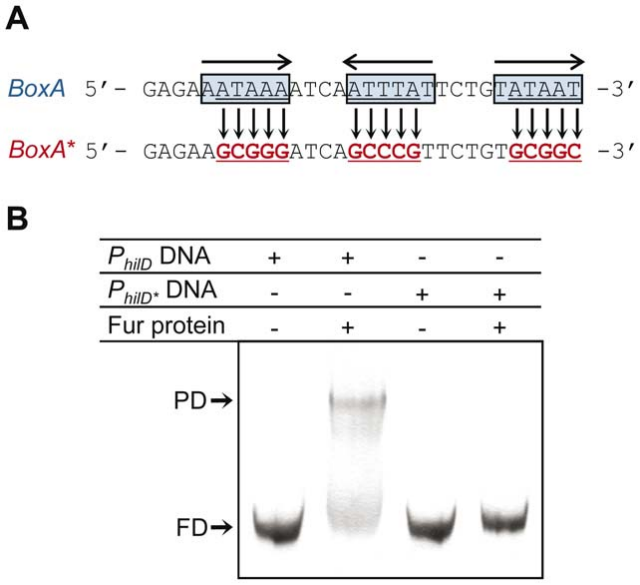
Fur·Mn^2+^ does not bind to a mutated BoxA (P_hilD__*_) **A.** Sequence of wild-type BoxA in *P_hilD_* and the mutated BoxA* variant in P_hilD*_. The three subsites of the Fur box and their relative orientation are also indicated. **B**. EMSA using 20 nM *P_hilD_* or P_hilD*_ DNA and 50 nM Fur·Mn^2+^. The presence or absence of a component is indicated inside the table by + or −, respectively.

To test the contribution of the AT-rich upstream operator (BoxA) to Fur recognition of *P_hilD_* DNA, a mutant BoxA DNA (P_hilD*_) was constructed and then used in EMSA experiments (Figure 4). As expected, *S. enterica* Fur specifically recognized *P_hilD_* DNA with wild-type BoxA in the upstream region but failed to bind P_hilD*_ carrying mutations in the three subsites of the Fur box region (Figure 4B).

### Fur activates *hilD* gene expression *in vivo*

**Figure 5:**
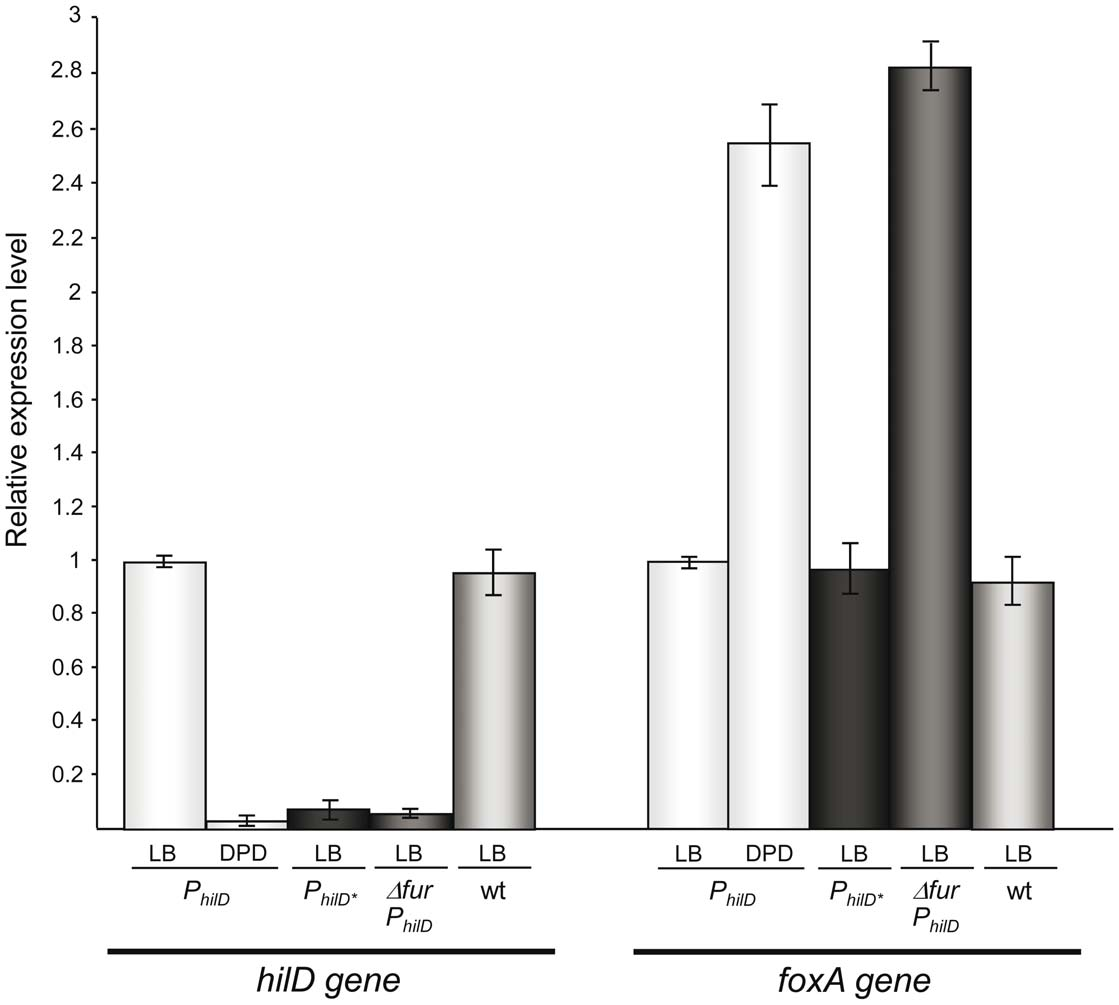
Relative *hilD* mRNA levels in several bacterial strains and iron concentrations. Relative *hilD* mRNA were estimated by qRT-PCR in *P_hilD_* (UA1888), P_hilD*_ (UA1889), *Δfur P_hilD_* (UA1890), or wild-type (SV5015) strains grown in high (LB) or low (DPD) iron concentration, as described in the Materials and Methods section. To demonstrate that *P_hilD_* utilization is not affected by the upstream Kan^R^ cassette, the *hilD* mRNA level in the wild-type SV5015 strain (wt) is also shown. The expression level of *foxA*, which is repressed by Fur [56], in all genetic backgrounds and conditions was determined as a control. For each condition, the relative gene expression levels were calculated as the ratio of each relative mRNA concentration with respect to that obtained in the isogenic wild-type strain (*P_hilD_*) and normalized to that of the *S. enterica recA* gene. The mean value from three independent experiments (each in triplicate) is shown.

To determine whether Fur increases *P_hilD_* utilization *in vivo*, the transcription level of *hilD* was assayed in several *S. enterica* strains. Wild-type *P_hilD_* and the P_hilD*_ variant, each with an upstream Kan^R^ cassette, were integrated into their native locus, leading to strains UA1888 and UA1889, respectively (Table 1). It is worth noting that the mutations in BoxA* (P_hilD*_, Figure 4A) were located upstream of the HilD, HilC, RstA [12], and RNA polymerase (RNAP) binding sites ([12], Figure 1A). Also, the presence of the Kan^R^ cassette upstream of BoxA did not modify *hilD* expression, since the transcription level of this gene was similar to that obtained using the SV5015 wild-type strain (Figure 5).

The *hilD* mRNA levels were analyzed by qRT-PCR and normalized with respect to the *recA* gene, which is not associated with the Fur regulon [64]. As a control, expression of the *foxA* gene, previously shown to be Fur repressed [56], was also measured under the same growth conditions. There was no evidence that the P_hilD*_ mutation affected *foxA* expression (Figure 5). In the absence of Fur or when iron was scarce (DPD addition), *foxA* expression levels increased (Figure 5), consistent with the ability of Fur·Fe^2+^ to repress *P_foxA_* utilization [56]. Under iron-saturated conditions, *hilD* mRNA level was ∼10-fold higher than under iron-limiting conditions (DPD addition) (Figure 5). Similar results were observed when 1.5 mM EDTA was added to the media as a chelator (data not shown). The fact that *hilD* expression level in the UA1888 (*P_hilD_*, DPD addition), UA1889 (P_hilD__*_), and UA1890 (Δ*fur*) strains were similar suggests that inactivation of BoxA (in P_hilD*_) had no effect on basal expression of the gene.

Under iron-saturated conditions, the expression of *hilD* mRNA in the P_hilD*_ mutant derivative was similar to that measured under iron-limiting conditions (Figure 5), suggesting that *P_hilD_* activation does not occur by a mechanism involving transcriptional de-repression. In the P_hilD*_ mutant derivative, *hilD* mRNA levels were also similar to those obtained with the wild-type *P_hilD_* in a null Fur mutant strain (Figure 5), implying that a wild-type BoxA sequence *in cis* is necessary for the increased accumulation of *hilD* mRNA.

There is a caveat to these findings, however, as there was no decrease in *hilD* expression in the absence of both HilD and Fur proteins, determined using transcriptional fusions [23]. This was confirmed by qRT-PCR experiments using the UA1892 (*Δfur ΔhilD*) strain, in which, as expected, *hilD* expression was increased by a factor of 1.2±0.18 with respect to the wild-type strain. Nonetheless, even though the absence of Fur or a decrease in the iron concentration resulted in a clear reduction in *hilD* expression (Figure 5), both HilD and Fur appear to be necessary for full *in vivo* expression of the gene. This complex response might be explained by the fact that only when the HilD protein reaches a significant threshold it is able to activate the expression of *hilA* and further induce its own expression [69].

### Fur·Mn^2+^ activates *hilD* expression *in vitro*

**Figure 6:**
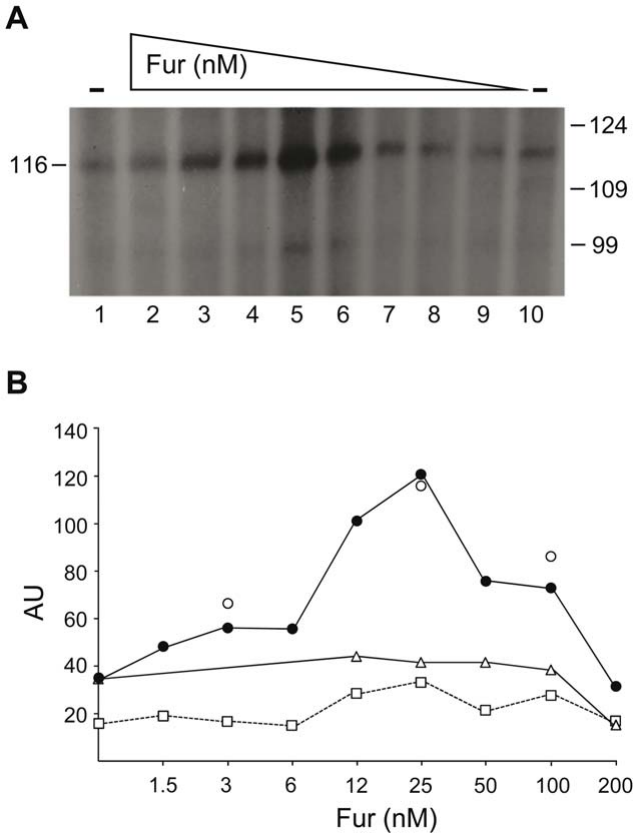
*In vitro* transcription of *P_hilD_* in the presence of Fur protein. **A**. Linear *Hin*dII-cleaved pUA1111 DNA (10 nM) containing *P_hilD_* was pre-incubated with increasing concentrations of Fur (1.5–200 nM). Transcription reactions, performed in the absence or presence of increasing concentrations of Fur, were initiated by adding RNAP and 0.5 mM of each rNTP (with [α-^32^P]-rUTP). The reactions were incubated for 60 min at 37°C. Transcripts generated *in vitro* from the *P_hilD_* template DNA were separated in 6% dPAGE. The *in vitro* transcripts from *P_hilD_* and their lengths are shown. The sizes of the markers are indicated. **B**. Linear *Hin*dII-cleaved pUA1111 or pUA1112 DNA (10 nM) containing *P_hilD_* or P_hilD*_ was pre-incubated with increasing concentrations of Fur (1.5–200 nM). Relative mRNA synthesis from *P_hilD_* (filled circles) and P_hilD*_ (empty triangles) in the absence or presence of increasing Fur was compared and is denoted in arbitrary units (AU). Also, the pUA1111 vector containing *P_hilD_* (*P_hilD_* sc) (open squares) and the pGEM®-T plasmid vector promoter (*P_v_*) (empty circles) were used under the same conditions as supercoiled DNA and the non-specific control, respectively.

To address whether the presence of Fur·Mn^2+^ is sufficient to activate *P_hilD_* utilization, *in vitro* transcription experiments with linearized *P_hilD_* (pUA111) or P_hilD*_ (pUA112) DNA were performed (Figure 6). A single transcript band was obtained with *Hin*dII-linearized DNA, indicating that *hilD* is expressed from a single promoter (Figure 6A, lane 1). The length of the transcript was in full agreement with transcripts initiated at *P_hilD_*, as determined by primer extension [12], confirming that the 116-nt transcript was the genuine transcript from *P_hilD_*. Minor transcripts with small molecular masses were attributed to RNAP pausing sites since no obvious promoter sequences could be predicted in the putative upstream regions.

In the presence of Mn^2+^ and the absence of Fur, steady-state levels of the 116-nt transcripts from *P_hilD_* and P_hilD*_ were similar (37±3 arbitrary units, AU), suggesting that the BoxA mutations did not affect P_hilD*_ utilization (Figure 6B). In the presence of Mn^2+^, *P_hilD_* utilization, or accumulation of the 116-nt transcript, increased with increasing Fur concentration, with an optimum reached when ∼ 2.5 Fur dimers/DNA molecule were added (123±5 AU) (Figure 6A). Similar levels of *P_hilD_* utilization were obtained using supercoiled DNA (*P_hilD_*sc) as template, ruling out any topological requirement for transcription activation (Figure 6B). At sub-saturating Fur·Mn^2+^ concentrations, *P_hilD_* utilization increased by more than 3-fold over the control without Fur·Mn^2+^ (Figure 6). However, the addition of Fur·Mn^2+^ did not significantly increase the levels of transcription of an unrelated promoter (*P*v) (27±5 AU) (Figure 6B).

P_hilD*_ utilization was not significantly increased at Fur·Mn^2+^ concentrations equal to or higher than those required to activate this promoter (Figure 6B). As seen in Figure 6B, Fur·Mn^2+^, at sub-saturating or half-saturating concentrations, interacted with BoxA, suggesting that the protein facilitates RNAP utilization of the *P_hilD_* region. It is therefore likely that Fur·Mn^2+^ acts as a transcriptional activator of *S. enterica hilD* expression. Since transcription activation was not observed when BoxA was inactivated by mutations (P_hilD*_), half-saturating Fur·Mn^2+^ concentrations are apparently necessary for transcription activation of the *hilD* promoter *in vitro*. However, in the presence of ∼ 20 Fur dimers/DNA molecule, similar activation was not observed (Figure 6B). Since ∼ 20 Fur dimers/DNA molecule similarly did not affect the expression of P_hilD*_ or an unrelated promoter (*P_v_*) (Figure 6), a contaminant RNase or any other non-specific effect can be ruled out as responsible for the reduced RNA synthesis at constant Mn^2+^ and higher Fur concentrations. It could be hypothesized that Fur·Mn^2+^ prevents *P_hilD_* activation based on its reported cooperative spreading. Thus, nucleoprotein assembly along the promoter region may interfere with, rather than stimulate, the interaction of RNAP with *P_hilD_*, returning *hilD* expression to its basal level.

## Discussion

We show that the *S. enterica hilD* gene, whose product is the most important regulator of the HilA activator and therefore of SPI1 T3SS expression, contains a Fur binding site (BoxA) in the upstream region of *P_hilD_* (−191 to −163). Fur, in its metal-bound form, bound with high affinity to BoxA in *P_hilD_* DNA but not to the BoxA mutant variant (BoxA*) in P_hilD*_. *In vivo* and *in vitro* experiments revealed that Fur bound to the upstream element of *P_hilD_* activated *hilD* expression, but did not activate transcription from P_hilD*_. Thus, metal-bound Fur appears to be a direct transcriptional activator of the *S. enterica hilD* gene. Being this the first evidence of Fur acting directly as an activator in this bacterium.

In most bacterial species, Fur·Fe^2+^ is a transcriptional repressor, binding to cognate sites within position −35 to +12 in the promoter region [33], [47], [57], [70]–[72]. In these cases, transcription of the target promoters is blocked by steric hindrance rather than by preventing transcription elongation [46], [66], [73]. Fur·Mn^2+^ bound to BoxA in *P_hilD_*, which is situated upstream of HilD, HilC (see Figure 1A [12]), or even RtsA binding sites [21], activates RNAP utilization of *P_hilD_*. A similar upstream location of the Fur cognate site has been described for Fur-activated genes in other microorganisms [46], [47], [51], [74], [75], suggesting a close relationship between the location of the Fur binding site in the promoter of the controlled gene and its role as an activator.

The majority of transcriptional activators, upon binding to their cognate upstream element adjacent to the core RNAP sites, either drive the recruitment of the latter to the target promoter or alter the conformation of the promoter DNA to facilitate RNAP loading [76]–[79]. Here we provide the first evidence of a direct mechanism of *S. enterica hilD* transcription activation by showing that Fur·Mn^2+^ binds to a distal upstream-activating sequence. We thus propose that Fur·Fe^2+^ is sufficient, *in vitro*, to increase RNAP recruitment. It is unlikely that Fur·Fe^2+^ alone alters the DNA conformation, because DNase I hypersenstive sites were not observed. There are not evidences for Fur·Mn^2+^ and RNAP interaction and activation via a looping mechanism, but Fur·Mn^2+^ bound to its target site are sufficient for increased *P_hilD_* utilization. It can be envisaged, however, that the HilD regulation *in vivo* is complex as suggested the *hilD* expression results obtained in the double *hilD fur* mutant and the fact that RtsA, HilC and HilD recognize a sequence downstream Fur and apparently antagonize H-NS- and Hha-mediated repression, suggesting that these complex control region (−120 to −57) laid within BoxA (−191 to −163) and the core promoter region (−60 to + 10) [21], [23].

The expression of SPI1 is known to be modulated by environmental signals, which indirectly control *hilD* transcription [3]. Among these signals, a relationship between extracellular iron concentrations and SPI1 expression has been suggested [23] and an indirect association of Fur and *hilA* expression through H-NS described [31]. In addition, several reports have shown that low-oxygen concentrations, such as those present in the intestinal lumen, increase SPI1 expression [4], [16], [80]. Free iron is scarce inside the host, but Fe^2+^ is abundant in the intestinal lumen, where it is efficiently absorbed by intestinal epithelial cells [28]. Fur does not sense oxygen concentrations directly but is instead able to monitor the redox signal via the equilibrium between Fe^2+^ and Fe^3+^ [81]. It should be noted that the Fur·Fe^3+^ complex is not functional and that, as Fe^3+^ is insoluble, it cannot be translocated inside the cell [33], [47].

**Figure 7:**
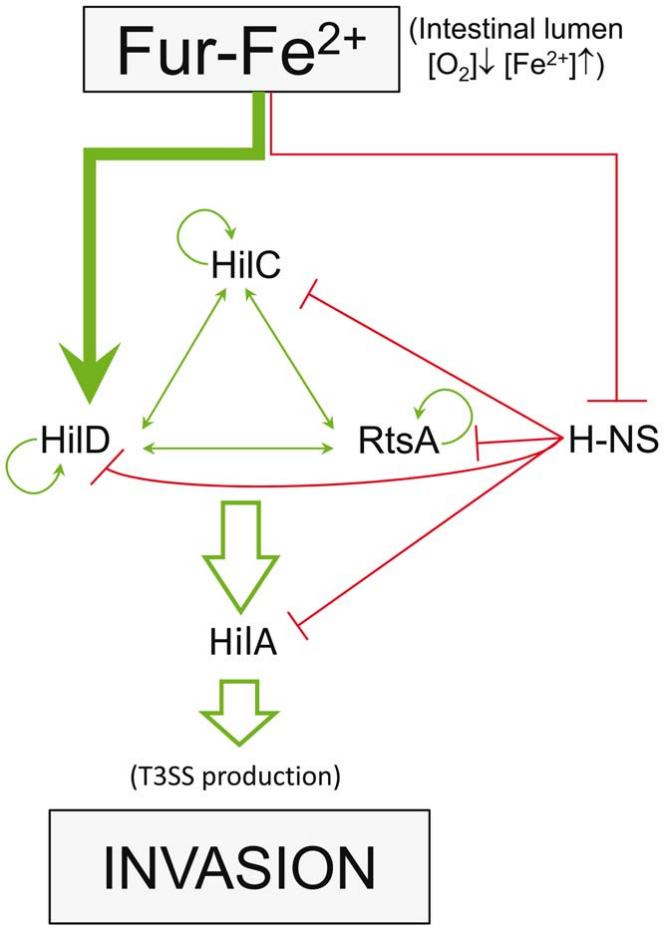
Scheme of SPI1-Fur mediated regulation. A Fur SPI1 regulation model after Ellermeier et al. [23] and Troxell et al. [31]. Green arrows indicate a direct activation effect; red lines correspond to direct repression. The thick green arrow shows the herein described pathway of direct activation by Fur of *hilD* gene expression. H-NS control of the *hilD, hilC, rtsA* and *hilA* promoters is also shown [21], [22], [31].

This work describes the direct activation of SPI1 by Fur through its interaction with an upstream region on *P_hilD_* and thus adds new information to Fur SPI1 regulation models [21], [23], [31] (Figure 7). Taken together, our data shed light on the role of Fur in SPI1 control. Specifically, Fur is able to control, either directly (in the case of HilD) or indirectly through H-NS (in *hilA, hilD, hilC,* and *rtsA*), all the main regulators of SPI1. These results strengthen the relationship between *S. enterica* invasiveness and both iron and oxygen concentrations inside the host. Accordingly, the expression of *hilD*, *hilA, hilC*, and *rtsA*, and consequently that of T3SS1, should be stimulated once *S. enterica* reaches the epithelial surface, where iron concentrations are high and those of oxygen low. T3SS expression has been reported as essential for epithelial invasion [5]. Once Fe^2+^ levels decrease or those of O_2_ increase, the number of Fur·Fe^2+^ complexes should diminish markedly, as should T3SS, which is no longer needed and must remain silent in subsequent steps of the infection process [5]. This sequence of events is supported by a previously published report in which, the epithelial invasiveness of Fur-defective mutants was shown, in an acid-sensitive independent manner, to be lower than that of the wild-type strain [82]. Direct regulation by Fur of *hilD* expression would allow rapid signal transduction once the Fe^2+^ concentration increases, situating *P_hilD_* in a similar hierarchic position as sRNAs that indirectly control other genes positively regulated by Fur.

## Supporting Information

Figure S1 **A**. SDS-PAGE showing the Fur purified protein. Lanes 1 and 2 correspond to non-induced and IPTG-induced cell crude extracts of BL21(DE3)pLys containing the *S. enterica fur* gene cloned in the pET15b vector. Lane 3 is the purification fraction containing the Fur native protein after thrombin digestion. **B**. Scheme of *P_foxA_* indicating the location of the Fur binding site in blue. The ATG start codon is indicated in bold. **C**. EMSA performed using DIG labeled *P_foxA_* probe (20 nM) and the purified Fur protein at increasing concentrations (2.5, 12.5, 50, and 187 nM). Lane (-) indicates the mobility of the DNA probe without Fur in the binding mixture.
(TIF)

Figure S2 Fur-Mn^2+^ binds with high affinity to *P_hilD_* DNA. The 375-bp [a-32P]-*NcoI-HindII* DNA (2 nM) fragment containing *P_hilD_* was incubated with increasing Fur concentrations (3–400) for 15 min at 37°C in buffer A (50 mM Bis-Tris/borate buffer pH 7.5, 5% glycerol, 10 mM MgCl_2_, 1 mM MnCl_2_) or E (50 mM Bis-Tris/borate buffer pH 7.5, 5% glycerol, 1 mM MgCl_2_, 0.1 mM MnCl_2_). The absence of a component is indicated by -; FD, protein-free *P_hilD_* DNA; IC, intermediate complexes; PD, protein-DNA complexes.
(TIF)

Figure S3 qRT-PCR assays of *recA* expression in the different genetic backgrounds used in this work. For each condition, the relative *recA* expression levels were calculated as the ratio of its mRNA concentration with respect to that obtained in the isogenic wild-type strain (*P_hilD_*) and normalized to that of the *S. enterica* 16S RNA. The mean value from three independent experiments (each in triplicate) is shown.
(TIF)

Table S1 Oligonucleotides used in this work.
(DOC)
