# Supplementary figures and images for: Fur Activates the Expression of Salmonella enterica Pathogenicity Island 1 by Directly Interacting with the hilD Operator In Vivo and In Vitro
Source: PLoS One. 2011 May 6;6(5):e19711. doi: 10.1371/journal.pone.0019711 (PMC3089636; doi:10.1371/journal.pone.0019711)

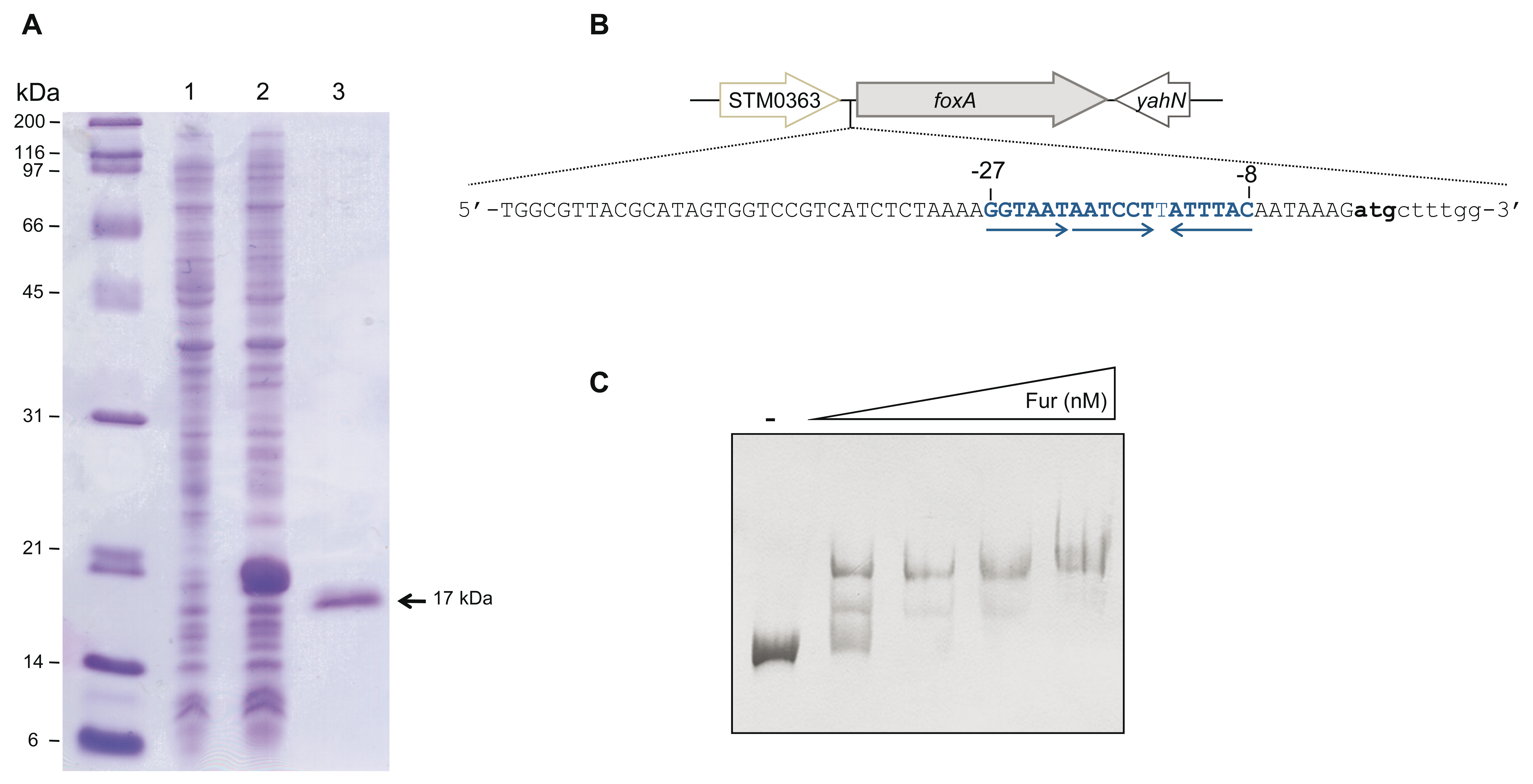

Supplement: Figure S1 — A. SDS-PAGE showing the Fur purified protein. Lanes 1 and 2 correspond to non-induced and IPTG-induced cell crude extracts of BL21(DE3)pLys containing the S. enterica fur gene cloned in the pET15b vector. Lane 3 is the purification fraction containing the Fur native protein after thrombin digestion. B. Scheme of PfoxA indicating the location of the Fur binding site in blue. The ATG start codon is indicated in bold. C. EMSA performed using DIG labeled PfoxA probe (20 nM) and the purified Fur protein at increasing concentrations (2.5, 12.5, 50, and 187 nM). Lane (-) indicates the mobility of the DNA probe without Fur in the binding mixture. (TIF) [file pone.0019711.s001.tif]

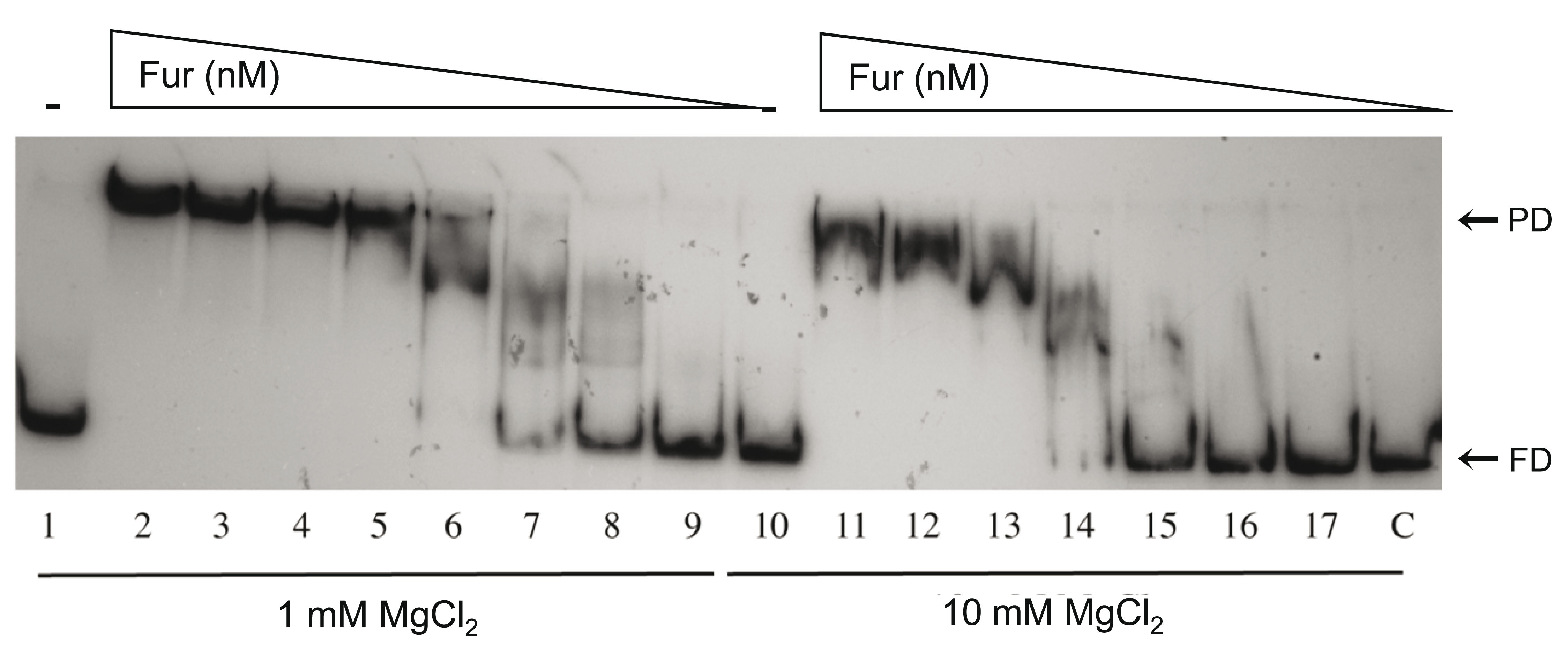

Supplement: Figure S2 — Fur-Mn2+ binds with high affinity to PhilD DNA. The 375-bp [a-32P]-NcoI-HindII DNA (2 nM) fragment containing PhilD was incubated with increasing Fur concentrations (3–400) for 15 min at 37°C in buffer A (50 mM Bis-Tris/borate buffer pH 7.5, 5% glycerol, 10 mM MgCl2, 1 mM MnCl2) or E (50 mM Bis-Tris/borate buffer pH 7.5, 5% glycerol, 1 mM MgCl2, 0.1 mM MnCl2). The absence of a component is indicated by -; FD, protein-free PhilD DNA; IC, intermediate complexes; PD, protein-DNA complexes. (TIF) [file pone.0019711.s002.tif]

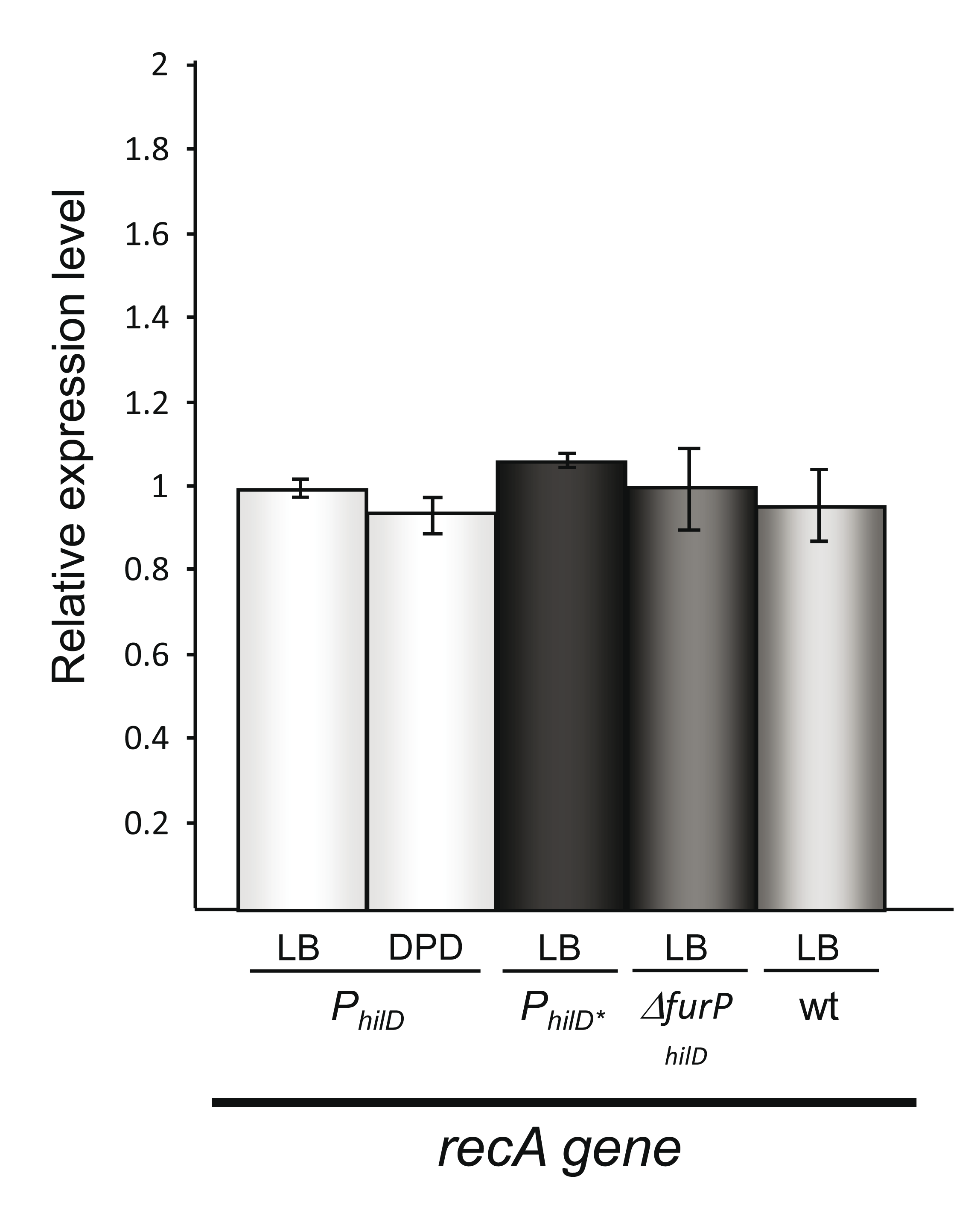

Supplement: Figure S3 — qRT-PCR assays of recA expression in the different genetic backgrounds used in this work. For each condition, the relative recA expression levels were calculated as the ratio of its mRNA concentration with respect to that obtained in the isogenic wild-type strain (PhilD) and normalized to that of the S. enterica 16S RNA. The mean value from three independent experiments (each in triplicate) is shown. (TIF) [file pone.0019711.s003.tif]
