# Supplementary material for: Fur Activates the Expression of Salmonella enterica Pathogenicity Island 1 by Directly Interacting with the hilD Operator In Vivo and In Vitro
Source: PLoS One. 2011 May 6;6(5):e19711. doi: 10.1371/journal.pone.0019711 (PMC3089636; doi:10.1371/journal.pone.0019711)
Supplement: Table S1 — Oligonucleotides used in this work. (DOC) [file pone.0019711.s004.doc]

Fur activates the expression of *Salmonella enterica* pathogenicity island 1 by directly interacting with the *hilD* operator *in vivo* and *in vitro*

Laura Teixidó^1^, Begoña Carrasco^2^, Juan C. Alonso^3^ Jordi Barbé^1^ and Susana Campoy ^1*^

^1^Departament de Genètica i de Microbiologia, Facultat de Biociències. Universitat Autònoma de Barcelona. 08193 Bellaterra, Spain, ^2^Area de Microbiología, Facultad de Medicina, Universidad de Oviedo, 33006 Oviedo, Spain, and ^3^Centro Nacional de Biotecnología, CSIC, 28049 Madrid, Spain.

Supplementary Table S1**.** Oligonucleotides used in this work

| Name | Sequence^a^ | Position^b^ | Application |
| --- | --- | --- | --- |
| *fur*NdeI | CATATGACTGACAACAATACCGC | 1 | 5’-end primer for *S. enterica* *fur* gene |
| *fur*BamHI | GGATCCTTATTTAGTCGCGTCATCGTGC | 453 | 3’-end primer for *S. enterica* *fur* gene |
| *P_hilD_*up | GCAGCAGATTACCGCACAGG | -282 | 5’-end primer to obtain FrgA or FrgD |
| *P_hilD_* dw | AGTTATCTGCGGCAGGACGC | 55 | 3’-end to obtain FrgA, FrgB or FrgC |
| *P_hilD_* -205 | TGTATAATGCGTCTCAACAC | -205 | 5’-end primer to obtain FrgB |
| *P_hilD_* -51 | TCAGTAGGATACCAGTAAGG | -51 | 5’-end primer to obtain FrgC |
| *P_hilD_* -158 | CCAATGGGGATGATGGTTC | -158 | 3’-end primer to obtain FrgD |
| *P_hilD_*up | AGGGATTCCTGATGAAAATAGAATGAAAAGTGAGAAATAAAATCAATTTATTCTGTATAATGCGTCTCAACACATATTAAAAGAACCATCATCCCCATTG | -258 | 5’-end primer to obtain *P_hilD_* |
| *P_hilD_*dw | CAATGGGGATGATGGTTCTTTTAATATGTGTTGAGACGCATTATACAGAATAAATTGATTTTATTTCTCACTTTTCATTCTATTTTCATCAGGAATCCCT | -158 | 3’-end primer to obtain *P_hilD_* |
| *P_hilD*_* up | AGGGATTCCTGATGAAAATAGAATGAAAAGTGAGAA**GCGGG**ATCA**GCCCG**TTCTGT**GCGGCG**CGTCTCAACACATATTAAAAGAACCATCATCCCCATTG | -258 | 5’-end primer to obtain *P_hilD_** |
| *P_hilD*_*dw | CAATGGGGATGATGGTTCTTTTAATATGTGTTGAGACG**CGCCGC**ACAGAA**CGGGC**TGAT**CCCGC**TTCTCACTTTTCATTCTATTTTCATCAGGAATCCCT | -158 | 3’-end primer to obtain *P_hilD_** |
| *P_hilD_*P1 | TAGTTTAAGCCCCAATGGGGATGATGGTTCTTTTAATATGTGTTGAGACGCATTATACAGAATAAATTGATTTTATTTCT*gtgtaggctggagctgcttc* | -145 | P1 primer for UA1888 construction |
| *P_hilD_*P2 | ATGCGATGTCTGTCGTTCTCGATAGCAGCAGATTACCGCACAGGACACAGGGATTCCTGATGAAAATAGAATGAAAAGTG*atgggaattagccatggtcc* | -304 | P2 primer for UA1888 and UA1889 construction |
| *P_hilD*_*P1 | TAGTTTAAGCCCCAATGGGGATGATGGTTCTTTTAATATGTGTTGAGACGC**GCCGC**ACAGAA**CGGGC**TGAT**CCCGC**TTCT*gtgtaggctggagctgcttc* | -145 | P1 primer for UA1889 construction |
| *hilD*P1 | CTCCGACTCCCGGAGAGTTATGAGATCATCCTTAGCTCGGCTCAGATAACGTTAAAGGAGCGCGTTTACAACATTATATC*gtgtaggctggagctgcttc* | 561 | P1 primer for UA1891 construction |
| *hilD*P2 | TAGTTTTAAAATATTTTTTGAAACATTGAATGAAGTAGGACGTGCTATCATAACCACATTTTAATGCTACAGCATTAACA*catatgaatatcctccttag* | 895 | P2 primer for UA1891 construction |
| *hilD*RTup | GAGATACCGACGCAACGACT | 304 | 5’-end primer for *hilD* gene RT-PCR analysis |
| *hilD*RTdw | AAGCAGGAACAGCAGAAAAT | 558 | 3’-end primer for *hilD* RT-PCR analysis |
| *recA*RTup | TGGCTATCGACGAAAACAAACA | 2 | 5’-end primer for recA RT-PCR analysis |
| *recA*RTdw | TTCCGCATCGATAAACGCACAG | 270 | 3’-end primer for *recA* RT-PCR analysis |
| *foxA*RTup | TAGCGCCGCCGTGTATCGTA | 1591 | 5’-end primer for *foxA* gene RT-PCR analysis |
| *foxA*RTdw | ATGCCGGAGCCCAAAGTCAG | 1871 | 3’-end primer for *foxA* RT-PCR analysis |

^a^ Restriction endonuclease recognition sites are underlined. Nucleotide substitutions in the Fur box are marked in bold. P1 and P2 sequences, homologues to the pKD4 plasmid, are represented in lower case italics.

^b^ Position of the 5’ end of the oligonucleotide with respect to the translational start point of the coding gene.
